# Senescence versus apoptosis in chemotherapy

**DOI:** 10.18632/oncotarget.3114

**Published:** 2015-01-30

**Authors:** Stéphane Ansieau, Guillaume Collin

**Affiliations:** Centre de Recherche en Cancérologie de Lyon, Inserm UMR-1052, CNRS UMR-5286, Université de Lyon I, Centre Léon Bérard, Lyon, France

Colorectal cancer (CRC) is a leading cause of cancer death worldwide. Distal recurrences represent the dominant problem with this disease. Adjuvant chemotherapy has an established benefit but the administration of highly effective chemotherapy regimens has proven difficult. In their manuscript, Barbara Jonchere and colleagues used different colon cancer cell lines with the aim to unveil novel mechanisms of resistance to sn38 treatment [1], the active metabolite of irrinotecan, a topoisomerase I inhibitor routinely used in adjuvant therapy. Using a time-limited cell treatment to mimic the therapeutic protocol, they confirmed the consequent DNA damage induction and subsequent cell commitment to a senescence program. In support for the role of mitotic skipping in senescence induction [2], senescent cells were confirmed to be tetraploid. Strikingly, ten days post-treatment, from among the senescent cells emerged proliferating diploid cells. These cells display a transformation potential as assessed in a soft-agar colony-formation assay. Where do these cells originate from? Although parental cells failed to generate colonies on agar, it remains difficult to exclude that these clones were present in the parental cell lines (with an average doubling time of 14h, 14 days of culture would mean a 25 fold enrichment). Cancer stem cells (CSC) are known to associate with relapse. However, enrichment in stemness markers including Lgr5, CD133, CD44 and ALDH1 activity was not observed, so excluding CSC enrichment. As an alternative, one could imagine that resistance would be acquired in the time course of treatment. Escape from therapeutic treatments has often been associated with an epithelial-to-mesenchymal transition (EMT). This cellular transdifferentiation mechanism associates with the reacquisition of some stem cell-like properties and multidrug resistance [3]. To evaluate such a possibility, effects of salinomycin, a potassium channel inhibitor known to eradicate EMT-committed mammary epithelial cells [4], was assessed. Noticeably, while salinomycin was selected for its ability to differentially target epithelial and mesenchymal cancer cells (fully committed into an EMT process), its impact on partially reprogrammed cells (incomplete commitment into an EMT process) still remains poorly addressed. Furthermore, EMT is a heterogeneous process with the genetic background and the local microenvironment being the predominant variables. Whether salinomycin eradicates all EMT-committed cells is still an open question. Surprisingly, the authors demonstrated that salinomycin rather facilitated than impeded the emergence of resistant cells. This observation is intriguing and disturbing, with salinomycin being included in clinical trials.

**Figure 1:**
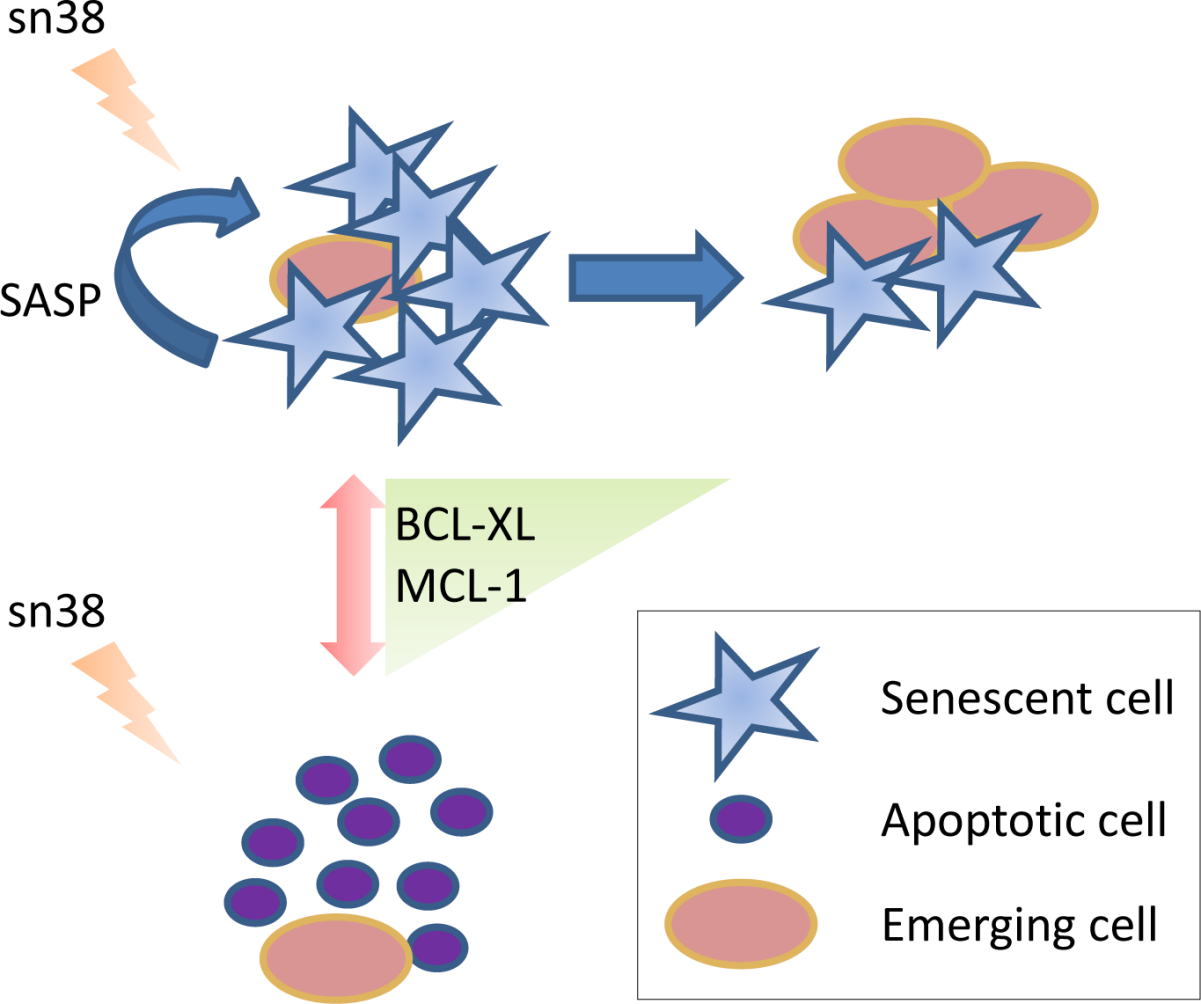
By favoring senescence instead of apoptosis, the BCL-XL/MCL-1 survival pathway facilitates the emergence of resistant cells

By sorting-out senescent and proliferating cells, the authors strikingly demonstrated that senescent cells were determinant in maintaining the proliferation and/or transformation potential of the emerging resistant cells (Figure 1). Although definitively growth-arrested, senescent cells are known to be metabolically active, secreting a set of cytokines and chemokines (senescence-associated secreted phenotype) that turn them into pro-inflammatory cells, facilitating tumor progression [5,6]. Whether the treatment of parental cells with supernatants from senescent cells is sufficient to afford them a resistance to sn38 still remains to be demonstrated. The authors lastly highlighted the role of the BCL-XL/MCL-1 survival pathway in the early response to the genotoxic treatment, which favored senescence induction rather than apoptosis. In line with this observation, they previously showed that these anti-apoptotic proteins play a pivotal role in the survival of intestinal cancer cells that escape from oncogene-induced senescence (OIS) [7]. Their contribution to the emergence of proliferative cells may of course differ depending on the origin of these cells. If these cells are present from the start, the two proteins might indirectly favor their proliferation by increasing the senescence messaging secretome. If cells escape from senescence (or pre-senescence) through reprogramming, the two anti-apoptotic proteins may contribute to their survival, as previously observed in cells that escape from OIS [7]. In conclusion, this study demonstrates the importance of cell fate (senescence versus apoptosis) in determining the effectiveness of a therapeutic treatment. In fact, favoring the destruction of senescent cells by the immune system may turn out to be essential in not only tumor clearance but also in the avoidance of recurrence.
